# Extracting information from the text of electronic medical records to improve case detection: a systematic review

**DOI:** 10.1093/jamia/ocv180

**Published:** 2016-02-05

**Authors:** Elizabeth Ford, John A Carroll, Helen E Smith, Donia Scott, Jackie A Cassell

**Affiliations:** ^1^ Division of Primary Care and Public Health, Brighton and Sussex Medical School, Brighton, UK; ^2^ Department of Informatics, University of Sussex, Brighton, UK

**Keywords:** electronic health records, review, text mining, data quality, case detection

## Abstract

**Background** Electronic medical records (EMRs) are revolutionizing health-related research. One key issue for study quality is the accurate identification of patients with the condition of interest. Information in EMRs can be entered as structured codes or unstructured free text. The majority of research studies have used only coded parts of EMRs for case-detection, which may bias findings, miss cases, and reduce study quality. This review examines whether incorporating information from text into case-detection algorithms can improve research quality.

**Methods** A systematic search returned 9659 papers, 67 of which reported on the extraction of information from free text of EMRs with the stated purpose of detecting cases of a named clinical condition. Methods for extracting information from text and the technical accuracy of case-detection algorithms were reviewed.

**Results** Studies mainly used US hospital-based EMRs, and extracted information from text for 41 conditions using keyword searches, rule-based algorithms, and machine learning methods. There was no clear difference in case-detection algorithm accuracy between rule-based and machine learning methods of extraction. Inclusion of information from text resulted in a significant improvement in algorithm sensitivity and area under the receiver operating characteristic in comparison to codes alone (median sensitivity 78% (codes + text) vs 62% (codes), *P* = .03; median area under the receiver operating characteristic 95% (codes + text) vs 88% (codes), *P* = .025).

**Conclusions** Text in EMRs is accessible, especially with open source information extraction algorithms, and significantly improves case detection when combined with codes. More harmonization of reporting within EMR studies is needed, particularly standardized reporting of algorithm accuracy metrics like positive predictive value (precision) and sensitivity (recall).

## INTRODUCTION

Information recorded in electronic medical records (EMRs), clinical reports, and summaries has the possibility of revolutionizing health-related research. EMR data can be used for disease registries, epidemiological studies, drug safety surveillance, clinical trials, and healthcare audits.

### Information recording in EMRs

In most EMRs there is the possibility for the clinician both to code their findings in a structured format and also to enter information in narrative free text. There are various nomenclatures for structuring or coding information; the most widely used are International Classification of Diseases version 10, ^1^ Systematized Nomenclature of Medicine – Clinical Terms, ^2^ and the International Classification of Primary Care. ^3^ Within multi-modal EMRs there are also laboratory, pathology, and radiology reports, admission and discharge summaries, and chief complaints fields, which are in unstructured or semi-structured text. The balance of recording by the clinician, between codes and narrative text, is likely to vary by institution, EMR system, department, disease type, and component of the record.

**Figure 1:**
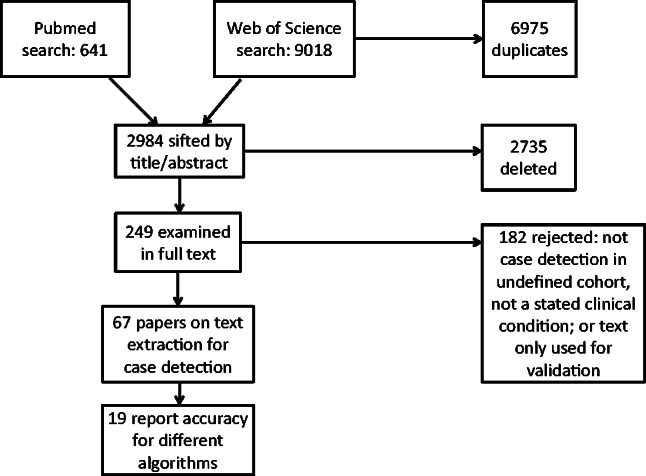
Flow diagram of study selection.

### Why do EMRs contain free text instead of being completely structured?

Clinicians experience a tension between choosing to code information and expressing it in text. ^4^ Among the main motivators for clinicians to code rather than use text is the increased ease of search, access, and retrieval. ^5^^,^^6^ A coded record allows the clinician to readily demonstrate that appropriate care has been provided, accurate diagnoses are made, and targets met. ^7^ This is especially important for billing after episodes of care, or for incentive based systems such as the National Health Service (NHS) Quality and Outcomes Framework in UK primary care. ^8^

Coded data can be analyzed and summarized easily and on a large scale, whereas free text cannot. In contrast to structured data, narrative text is highly variable, ^9^ but is more engaging, captures the patient’s narrative, can be told from different perspectives, and allows expression of feelings. ^10^ It is a better reminder for the clinician of the human encounter. ^7^

Additionally, clinicians have given a number of reasons why they find coding onerous; the choices available in coded data may be too limiting, and may not allow for the expression of nuances. ^11^ The process of finding and entering codes on the computer represents an additional cognitive load, ^5^ and may take longer than summarizing the consultation in text. ^6^ Free text may be chosen when no code precisely describes clinical findings, or when there is a need to give supporting evidence for a diagnosis or suspicion. ^12^ Clinicians use free text as a pragmatic solution to recording vague diagnoses or strange collections of symptoms, when diagnoses need qualification, and for psycho-social problems. ^7^ Text can summarize processes of deduction, and modal language can be used to convey a range of possible outcomes. Codes do not easily accommodate diagnostic uncertainty, so a patient may be labeled with a diagnosis prematurely or incorrectly. Similarly, a clinician may have a range of possible differential diagnoses, but only code the one that supports the choice of treatment. ^7^

### Why case-detection is central to EMR research

One key quality issue in research using data from EMRs is the precision of case-detection. Studies have shown that classification errors in the case identification process can considerably bias study findings. ^13^ If cases of the disease of interest are not well defined, then the conclusions drawn from the study will be of poor quality. Case-detection algorithms are created from several structured pieces of information, such as sets of diagnostic and prescription codes; existing examples include dementia, ^14^ stroke, ^15^ diabetes, ^13^^,^^16^ depression, ^17^ hypertension, ^18^ and rheumatoid arthritis. ^19–23^

To date, research using EMRs has mainly relied on coded information to define cases. Abstraction and analysis of the coded information is straightforward in comparison to abstraction of the text, which also requires anonymizing and annotating. As yet we have little understanding of how much information, and what type, is contained within unstructured sections of the record, and therefore how biases may arise from ignoring the content of the text. Adding in text may markedly improve *rates* and *accuracy* of case-detection when using EMRs for research. UK studies have shown that our understanding of the date of diagnosis, ^24^ and the number of symptoms prior to diagnosis ^12^^,^^25^ can change substantially when information extracted from free text is added to the coded information.

### Methods for extracting information from text

The volume of EMRs available means that human review of text is too time-consuming and labor intensive to be achievable in most studies. However, the automation of extraction of information from text makes the clinical information contained therein more accessible. Natural language processing (NLP) is a subfield of computer science concerned with intelligent processing of human language. For over 50 years computer scientists have developed algorithms to analyze natural language text, using either sets of hand-written rules or machine learning techniques. ^26^ However, adapting such algorithms to medical text has proved difficult, for two main reasons: 1) patient privacy and confidentiality issues, which create difficulties in obtaining suitable data to develop and test algorithms on ^27^^,^^28^ and 2) the nuances of medical text, which make it difficult to obtain reliable clinical results using standard processing techniques. ^29^^,^^30^ The majority of tools for analysis of text are trained on edited text genres such as newspaper articles or scientific papers. ^31^ While medical discharge summaries, diagnostic test reports, and letters may be written in standard English, consultation notes are hastily written, and do not go through an editing process. These notes are terse, with a telegraphic style and limited use of full sentence syntax; in particular, sentential subjects are very rare, and even finite verbs are uncommon. ^31^ Standard NLP tools make many errors when applied to clinical notes. It has often been necessary for a new NLP tool to be developed or adapted for each medical database, and even for each clinical question, when processing EMR free text. This is labor intensive, as it requires the tools to be tested on significant amounts of text already annotated by human experts.

### Aims

It is not clear how successful researchers have been in incorporating information extracted from EMR text into their case-detection algorithms, or how much of an improvement the addition of this information gives in comparison to codes alone. In the present study we aimed to review information extraction from EMR text for the stated purpose of case-detection for named clinical conditions.

In particular, we aimed to 1) systematically describe the methods of information extraction from text, 2) evaluate the current technical accuracy of information extraction algorithms, and 3) understand the additional benefits of using text for case-detection rather than structured data alone.

## METHODS

### Systematic search

Searches were conducted between July 2014 and July 2015 on PubMed and Web of Science (WoS), using search terms derived from Medical Subject Headings vocabulary (US National Library of Medicine): 1) “electronic health records” or “electronic medical records” or “electronic patient records” or “hospital records” or “personal health records” or “computerized patient records” or “computerized medical records” or “automated medical records” combined with 2) “free text” or “narrative” or “text mining” or “natural language processing.” No date constraints were placed on papers retrieved. These searches returned 641 articles from PubMed and 9018 from WoS, of which 6975 were duplications within WoS search results or between WoS and PubMed results. Following review of titles and abstracts, 249 papers were retained to examine in full text.

### Eligibility

To be eligible for this review, published research had to meet all of the following four criteria:

1. Primary research with full text published in English.
2. Information extracted from the text of EMR, medical letter, or medical report by any method.
3. Information extracted from text for stated clinical condition.
4. Stated purpose of information extraction was case-detection.

### Exclusion of papers

Papers were excluded in two stages; a title/abstract review (2735 excluded) and then a full text review of 249 papers (182 excluded). In total 67 papers met the eligibility criteria ( Figure 1 ). Major reasons for rejection were because papers focused on:

Describing or comparing EMR systems.
Generating text from EMR data.
Problem list or decision support development, clinical interventions delivered through EMRs.
Acceptability, satisfaction, barriers, or facilitators with EMR systems.
Technical details of information extraction with no stated clinical condition.
Cause of injury or event detected rather than clinical condition.
Extraction of information from text for purposes other than case-detection.
Extraction of characteristics of a defined population, rather than pure case-detection.
Text used only for case validation not detection.

### Extraction of information from studies

The full text of all studies was scrutinized and details were abstracted into a table ( Supplementary Data ). Wilcoxon signed rank tests were performed to compare extracted values of median accuracy of algorithms between studies, using IBM SPSS statistics 22.

### Assessment of algorithms

The most rigorous method for assessing the accuracy of an algorithm is to compare its results against a gold standard. Most studies reported in this review assessed performance by means of manual review, unless noted otherwise. Method of assessment was not an eligibility criterion for inclusion of a study; studies were included even if they reported no assessment of algorithms. If any of the following measures was stated in the study it was extracted and reported here, and studies reporting any of these measures were included in the technical accuracy section of the results. Measures included:

1) **Sensitivity (Recall)** measures the proportion of actual positives that are correctly identified as such (e.g., the percentage of sick people who are correctly identified as having the condition).
2) **Specificity** measures the proportion of negatives that are correctly identified as such (e.g., the percentage of healthy people who are correctly identified as not having the condition), and is complementary to the false positive rate (1 – False Positive Rate).
3) **Positive predictive value (precision) and negative predictive value** (PPV and NPV, respectively) are the proportions of positive and negative results in tests that are true positive and true negative results. These values are dependent on the prevalence of the condition in the population, so a low prevalence condition may give rise to a low PPV despite high sensitivity and specificity.
4) **F-measure** : In informatics, the positive predictive value is called precision, and sensitivity is called recall. The F-score can be used as a single measure of performance of the test and is the harmonic mean of precision and recall:
  $$F=2X\frac{\text{precision}\;\text{x}\;\text{recall}}{\text{precision+recall}}$$
5) **Area under the ROC (AUROC)** : The trade-off between sensitivity and specificity can be represented graphically as a receiver operating characteristic (ROC). The ROC curve illustrates the performance of a binary classifier system as its discrimination threshold is varied. When using normalized units, the area under the curve (often referred to as simply the AUC, or AUROC) is equal to the probability that a classifier will rank a randomly chosen positive instance higher than a randomly chosen negative one.

## RESULTS

The 67 studies included in this review were published between 2000 and 2015, with the majority from 2010 to 2015 (41 studies, 61%). The majority of studies used data that originated in the United States (US) (and were conducted by US teams; 57 studies, 85%). Data from the Netherlands was used in eight studies (12%; including two that incorporated data from Italy and Denmark). One study was conducted using data from Canada (1%), and one using data from Sweden (1%).

### Reasons for case-detection

The majority of studies (87%) gave a reason for wanting to detect cases from medical records. The most common reasons were to use them in further medical records research (29 studies, 43%). Other studies stated the purpose was for epidemic surveillance of infectious diseases (12 studies, 18%); for surveillance of indicators of cancer, diabetes, or hospital acquired infection to assist prevention (4 studies, 6%); for estimation of incidence of conditions in the population (5 studies, 7%); or for clinical trial recruitment (5 studies, 7%). Three studies stated they were seeking improvements in clinical decision-making (4%), and one study was populating a cancer registry (1%). Nine studies (13%) did not identify the purpose of case-detection.

### Conditions studied

Forty-one conditions were studied in the 67 studies, and four studies each ascertained two conditions. Conditions could be divided into four categories: chronic or noncommunicable diseases, infectious diseases, psychological disorders, and injuries or events ( Table 1 ).

**Table 1:** Types of Conditions Studied

Type of condition	No. of studies	Conditions included ( *N* studies if >1)
Chronic or noncommunicable conditions	42 (59%)	Obesity (7), cancer (4), rheumatoid and psoriatic arthritis (5), diabetes (3), inflammatory bowel disease (incl. celiac) (3), asthma (3), COPD (2), pancreatic cyst (2), heart failure (2) hypertension, angina pectoris, atrial fibrillation, disorders of sex development, multiple sclerosis, hepatobiliary disease, cataract, priapism, facial pain, peripheral arterial disease, coronary artery disease
Infectious diseases	18 (25%)	Acute respiratory infection (2), pneumonia (4) influenza or influenza-like illness (5) MRSA (2), gastrointestinal infection, genital chlamydia, chicken pox, fever, hospital acquired urinary tract infection
Psychological disorders	4 (6%)	Depression (2), binge eating disorder, bipolar disorder
Injuries and events	7 (10%)	Venous thromboembolism (2) acute myocardial infarction, upper GI bleeding, ischemic stroke, acute renal failure, acute orbital fracture

### Types of medical records

Thirty-seven studies drew on multiple sections of hospital EMRs, such as codes, prescriptions, laboratory or pathology reports, and clinical notes (55%). Other studies used a focused part of the hospital EMR: nine studies (13%) used hospital discharge summaries, five studies (7%) used imaging reports (X-ray or CT scans), three (4%) used the narrative portion of emergency department records, two (3%) used laboratory reports only, and one study used pathology reports (1%). Ten studies (15%) used primary care records that contained a mixture of structured fields (codes and prescriptions) and free text.

### Information extraction from text

There were three main types of information extraction: keyword search, rule-based algorithm, and machine learning algorithms. Sixteen studies (24%) used only a keyword search to extract information. Forty-five studies (67%) reported a rule-based NLP algorithm to extract information from text. An algorithm was categorized as rule-based if it combined a keyword search with any negation or context modifying module, although many algorithms were more sophisticated than this. Six studies (9%) used machine learning, Bayesian, or hybrid (rule-based + machine learning) approaches.

Several information extraction algorithms were used in more than one study. Studies used established NLP algorithms such as MedLEE (9 studies), ^32^^,^^33^ HITEx (4 studies), ^34^ cTAKES (5 studies), ^35^ Unstructured information management architecture (3 studies), ^36–38^ Topaz (2 studies), ^39^^,^^40^ Regenstrief extraction tool (REX; 2 studies), ^37^^,^^41^ and the KnowledgeMap concept identifier (2 studies). ^42^^,^^43^ Keyword search tools reported in more than one study included EMERSE (2 studies) ^44^ and the Unified Medical Language System (UMLS) search tool (2 studies). The most common structured output format of algorithms was the National Library of Medicine UMLS Metathesaurus of Concept Unique Identifiers, ^45^ which was used in 23 studies. NLP algorithms also output to the Systematized Nomenclature of Medicine Clinical Terms, Medical Subject Headings, and Hospital International Classification of Disease Adaptation codes.

Context modifiers and negation were assessed by several add-on algorithms, notably NegEx (5 studies), ^46^ and ConText (2 studies). ^47^ Medication information was extracted using MedEx (2 studies), ^48^ which produced RxNorm encoded medications, and FreePharma NLP (1 study). ^49^

### Case-detection algorithms (CDA)

After information from text was extracted, there were several different methods for reaching ascertainment of cases. Three studies manually reviewed the results of a keyword search, and four studies considered the presence of a single code or keyword to be sufficient for a case. The remaining 60 studies (90%) used an algorithm to detect cases. In 15 studies (23%) the same algorithm performed NLP and detected cases. In 16 studies (24%), a new algorithm was used to combine outputs of NLP using only textual information. In 29 studies (43%), the information from text was combined with codes, lab results, or medications to detect cases, using rule based, logistic regression, Bayesian, or machine learning models. The breakdown of algorithm types is shown in Table 2 .

**Table 2:** Types of Case-Detection Algorithms

Type of case-detection	No. of studies (%)	Detail
No additional algorithm (manual review of information)	3 (4)	
Single keyword or code sufficient to define case	4 (6)	
Same NLP algorithm as extracted info also detected cases (text only)	15 (23)	
New rule-based CDA (text only)	11 (16)	
Logistic regression or machine learning CDA (text only)	5 (4)	Logistic regression ^50^ ; decision tree ^51^ ; Bayesian network vs rule-based ^39^ ; naïve Bayes vs perception neural network ^52^ ; naïve Bayes ^53^
New rule-based CDA (combining text with codes, labs, or medication)	12 (18)	
Logistic regression CDA (combining text with codes, labs or medication)	8 (12)	
Machine learning algorithm (combining text with codes, labs, or medication)	6 (9)	Ripper ^54^
		Support vector machines (SVM) ^55^
		Decision tree, vs SVM vs Ripper vs metacost ^56^
		Naïve Bayes vs SVM vs random forest vs logistic regression ^57^
		Bayesian network model vs EM-MAP model ^40^
		Random forest ^58^
Comparison of rule based CDA with machine learning and logistic regression CDAs (combining text with codes, labs, or medication)	3 (4)	Rule based vs SVM vs random forest vs Ripper vs logistic regression ^59^
		Rule based vs logistic regression ^60^
		Rule based vs decision tree ^61^

### Technical Accuracy

Table 3 summarizes technical accuracy by type of case-detection algorithm and by medical condition. Fifty-six studies reported accuracy metrics for their algorithms and were grouped into three sets: 15 studies reported no additional CDA on top of the NLP algorithm extracting information from text ( Supplementary Table A ); 20 studies reported secondary rule-based case detection algorithms using combinations of different sources of text, or combining text, codes and medication ( Supplementary Table B ); and 21 studies reported probabilistic case detection algorithms (regression, Bayesian, or machine learning) combining different sources of text, or combining text, codes, and medication ( Supplementary Table C ). In order to ascertain whether technical accuracy was influenced by the type of condition, the median accuracy of algorithms is shown for conditions that are examined in more than one study (full details in Supplementary Table D with references). Table 3 shows no clear pattern of difference in accuracy by type of algorithm, nor much variability in performance by condition, with the exception of obesity, the ascertainment for which had lower than average performance, and for which the majority of studies were using a single source of data (hospital discharge letters in the i2b2 challenge ^62^ ).

**Table 3:** Median accuracy by algorithm type and condition

	No. of Studies	Sensitivity (Recall)	Specificity	PPV (Precision)	Negative predictive value	F measure	AUROC
**Algorithm type**							
Single algorithm for NLP and case detection	15	96.2	97.4	85.35	96.6	49	–
Rule-based secondary case detection algorithm	20	91.2	95.45	77.5	98.95	97.57	94.4
Probabilistic secondary case detection algorithm (Logistic Regression; Bayesian; machine learning)	21	80	95	86	95.4	77	94
**Condition**							
Respiratory infections	11	92.9	95.45	54	99.9	–	95.85
Bowel disease	4	79.45	94.45	57.5	100	–	87.5
Inflammatory arthritis	5	70	96	93.7	–	–	94.4
Cancer	3	93	92.9	95	–	93.5	–
Diabetes	2	96.2	98	–	–	98.65	–
Obesity	5	48.4	–	76.3	–	49	–
Mental health	3	73.1	90	87.85	96.6	–	80
MRSA	2	99.2	99.4	97.9	–	99	–
Cardiovascular	7	82	96	84.7	93	74.85	92.9

### Additional benefit of information extraction from text

The main benefit of extracting information from text was that case-detection was significantly improved. Table 4 shows selected accuracy metrics for 19 studies that reported direct comparisons of case-detection algorithms using codes only, text only, and/or a combination of codes and text. Medians were significantly higher in code/text-combined algorithms compared to codes-only algorithms for sensitivity (recall) ( *P* = .028) and AUROC ( *P* = .025), but not for PPV (precision) ( *P* = .066). There were no significant differences between accuracy of algorithms using codes only and text only.

**Table 4:** Accuracy of case-detection algorithms comparing codes and text

		Codes only			Text only			Combination of codes + text		
Study	Condition	Sensitivity (Recall)	PPV (precision)	AUROC	Sensitivity (Recall)	PPV (precision)	AUROC	Sensitivity (Recall)	PPV (Precision)	AUROC
Gundlapalli (2008) ^68^	Inflammatory bowel disease	27	50	64	86	43	90	100	40	99
Graiser (2007) ^69^	Lymphoma	42.9			90.0			81.2		
Valkhoff (2014) ^70^	Upper GI bleed									
	ICD-9 (ARS)		72							
	ICD-9 (HSD)		78							
	ICD-10 (Aarhus)		77			47				
	ICPC codes		21			22				
DeLisle (2013) ^71^	Pneumonia	52	52.8		74.8	63.6				
Li (2008) ^63^	Ischemic stroke	90			56					
Ludvigsson (2014) ^72^	Celiac	53.8			78.1					
Pakhomov (2007) ^53^	Angina	88			88					
Ananthakrishnan (2013) ^66^	Inflammatory bowel disease: Crohn’s			89						95
	Ulcerative colitis			86						94
Carroll (2012) ^42^	Rheumatoid arthritis	49	80	88				71	86	97
Liao (2010) ^73^	Rheumatoid arthritis	51	88		56	89		63	94	
Xia (2014) ^74^	Multiple sclerosis	76.4	91.6	93.7	75.8	91.4	94.1	82.7	92.1	95.8
DeLisle (2010) ^75^	Acute respiratory infection	79	31.5	88	88	18	94	73	52	86
Zheng (2014) ^76^	Acute respiratory infection	79	31	78	88	18	90	75	49	87
Carroll (2011) ^55^	Rheumatoid arthritis	78.1	93.2	95.5	68.8	91.8	89.5	85.8	93.7	96.6
Karnik (2012) ^57^	Atrial fibrillation	61.7	59.8		62.7	58		60	60	
Castro (2015) ^60^	Bipolar disorder		79			85				
McPeek (2013) ^43^	Venous thromboembolism		69		95	90				
Wu (2013) ^61^	Asthma	30.8	57.1		84.6	88.0				
Zeng 2006 ^77^	Asthma and COPD	72.5	82.3		76.7	82.3		92.4	87.4	
	**Median**	**61.7**	**72.0**	**88.0**	**78.1**	**73.0**	**90.0**	**78.1**	**86.0**	**95.4**

Some other studies reported other improvements in case finding with the addition of text. Friedlin et al. ^41^ reported that their NLP algorithm accurately identified three times as many methicillin-resistant Staphylococcus aureus (MRSA) positive blood cultures as their current electronic laboratory reporting system. Li et al. ^63^ reported that of 2609 cases detected, MedLEE found 1253 (48%) that were not retrieved by searching International Classification of Diseases version-9 codes. A further five studies reported an increase in the number of cases found by using text, including for cancer, ^64^ hypertension, ^65^ inflammatory bowel disease, ^66^ ischemic stroke, ^63^ and disorders of sex development in children. ^67^ These studies reported a statistically significant increase in cases ( *P* = .003), ^64^ a 7–12% increase in cases, ^65^^,^^66^ 226 patients being found using keyword search compared to 14 with manual search, ^67^ and 702 more patients found using text than with codes alone. ^63^

## DISCUSSION

This review of extracting information from the text of EMRs for case-detection has shown that text can contribute to case-detection of a wide range of conditions including infectious diseases, noncommunicable diseases, and acute events, as well as psychological conditions. However, differences in accuracy of case-detection using information from text compared to codes alone are not always reported explicitly or in a useful form.

The eligible studies suggest that the majority of work so far has been conducted in the United States; very few other countries are represented. The majority of data sources used in these studies were full multi-modal electronic hospital record systems and parts of these records, such as discharge summaries or pathology reports. The source of information is important to note as it affects the portability of the method of information extraction. Documents such as reports and clinical notes use a terse, telegraphic style where the grammatical rules of standard English are discarded in favor of concise information presentation, and where the recipient or reader already has good knowledge of common abbreviations and contractions. Discharge summaries or letters may use more standard English structures and therefore algorithms developed for non-medical text sources may be suitable.

The technical accuracy of algorithms extracting information from text, or combining text information with codes, was generally good but with some variability. Because of the wide range of possible measures of algorithm accuracy, many algorithms were not directly comparable to one another. Many studies reported algorithms with sensitivity and specificity (and related values) of over 90%. Different methods of information extraction were reported, ranging from manual review of records to both rule-based algorithms and probabilistic or statistically driven models using machine learning methods. No particular type of algorithm stood out as particularly better than any other. Accuracy also varied by condition, but no clear pattern was evident.

Some studies reported statistically on the additional benefit to case-detection of extracting information from text compared to codes alone and found there was a significant improvement in case-detection accuracy by incorporating information extracted from text. Given that only 19 studies reported these comparisons, with a large proportion of missing data due to inconsistencies in reporting, this finding needs to be confirmed in a larger pool of studies.

### What are the future directions for information extraction from EMR text?

There is no consensus in the literature of what is “good enough” for case-detection models or how much error is acceptable when ascertaining cases. If these algorithms were to be used for identifying patients for clinical trials, or for estimating service needs, a high standard of accuracy would be required. While sensitivities and specificities over 95% sound impressive, if we are looking for cases of a disease with a 1% prevalence, using a case-detection algorithm with a 98% sensitivity and a 97% specificity, the probability that a patient identified as a case really is a case (PPV) is only 25%, because of the high number of false positives. ^78^ Work is needed to understand better what constitutes appropriate and safe standards for identifying patients or outcomes for research by these methods.

Additionally, there appear to be two cultures of reporting: The consensus within the field of informatics is to report the measures *precision* , *recall* , and *F-measure* , whereas in medicine, the practice is usually to use *sensitivity* and *specificity* . Researchers in *biomedical* informatics understand that sensitivity is equal to recall and positive predictive value is equal to precision. Specificity on the other hand is not used in informatics outside the biomedical domain. To make it easier to compare results and draw conclusions from them, the two cultures must become more integrated. The aim should be for more standardized ways of reporting the accuracy of both information extraction and case-detection algorithms.

These studies used many different algorithms for information extraction from text, and in around half of studies, algorithms were specific to the individual study. Such algorithms take significant human effort and time to develop, requiring domain expertise, programming skills, and iterative evaluation and development. ^42^ Re-using existing algorithms and nomenclatures minimizes effort and ensures comparability with other studies. MedLEE was utilized across nine studies, HITEx in five studies, and CTAKES in seven. Two studies reported specifically on the portability of an NLP algorithm keyword search tool, ^42^^,^^67^ suggesting the porting was successful with minor moderations to the algorithms. Future research could also investigate which approaches give the best chance of portability of CDAs to different settings, conditions, and purposes.

Another approach for reducing the effort associated with extracting information from text would be to develop generalizable estimates of context effects. Some studies reported keyword searches, which do not require complex algorithms, and can be a cheap and quick method of extracting information from text. However, these searches pick up all incidences of keywords, not taking account of negation, uncertainty, or other contextual effects. If we were able to estimate that the influence of context effects or modifiers were small, we would be reassured that keyword searches were an adequate and pragmatic approach to extracting information from text.

Achieving anonymity or de-identification is another barrier to the use of text from EMRs. In general, data protection regulations state that only de-identified data can be released to researchers without the patient’s explicit consent. De-identification of structured records is fairly straightforward, but anonymizing free text is a much more difficult task, as patient identifiers may be located in any part of the text. Algorithms that automate the process of de-identification of text have been developed and are reviewed elsewhere. ^28^^,^^79^ If these algorithms perform well enough, they could be run at source—for example, within the clinical institution where identifiers are not a problem—and anonymize the text before EMRs are extracted for secondary purposes. A set of standards for safe and secure de-identification to protect patient privacy is needed, therefore, so that the accuracy of de-identification algorithms can be compared against these standards.

### Strengths and limitations of the current study

This study identified a good range of published papers on extraction of information from text in EMRs. We used two sensitive databases that covered both medical and informatics fields to pick up as many articles as possible. Once studies were retrieved from the search they were then scrutinized and chosen in a rigorous fashion. This means the selection process was likely to have favored specificity over sensitivity in terms of studies meeting eligibility criteria, but whether this will have affected our conclusions is not clear. It may have reduced the power to find differences between types of algorithms. Additionally, only studies published in English were used. We are aware that some NLP groups also publish in French and German, so future work may seek to incorporate these studies by searching in other languages.

As this is a wide literature, it was not possible to also include studies reporting on extracting characteristics of defined populations, although the methodologies used in these studies would have considerably overlapped with the studies reported. A further review may want to scrutinize other reasons for information extraction from EMR text, such as medication usage and adverse events, or the quality of care given. It is possible that text may contain more valuable information for some research purposes than others, and so the value of extracting information from text should be reviewed for a range of purposes and compared.

One limitation of the literature identified in the current study was the small number of studies explicitly comparing algorithms containing information extracted from text to other algorithms using structured data only—less than one-third of studies identified for this review. If consensus on reporting within studies can be achieved, especially within those comparing case detection methods, this review could usefully be repeated in a few years’ time and further results ascertained to support the inclusion of information from text in EMR research.

### Conclusions

A wide range of studies showed that information extracted from EMR text has been used to identify varied conditions with variable degrees of success. Most of the research has so far come from research groups in the United States using hospital-based EMRs. There is likely to be benefit gained from adding information extracted from text to case-detection algorithms in terms of improved sensitivity and specificity, although numbers of studies are too small to make firm conclusions. There is no standardization in the reporting of the performance of the algorithms, which makes comparison of studies difficult. Researchers in the field would benefit from more standardized reporting of algorithm performance, such as always reporting sensitivity (recall) and PPV (precision), and from working towards making information extraction methods and their outputs more compatible and comparable between studies.

## FUNDING

This work was supported in part by the Wellcome Trust, grant number 086105/Z/08/Z.

## COMPETING INTERESTS

The authors have no competing interests to declare.

## Supplementary Material

Supplementary Data
