# Supplementary material for: Extracting information from the text of electronic medical records to improve case detection: a systematic review
Source: J Am Med Inform Assoc. 2016 Feb 5;23(5):1007–15. doi: 10.1093/jamia/ocv180 (PMC4997034; doi:10.1093/jamia/ocv180)
Supplement: Supplementary Data [file ocv180_Supplementary_Data.zip › Appendix_1.docx]

| **Study Name** | **Exact aim** | **Condition** | **Country** | **Type of data** | **Extraction of information from text** | **Case detection algorithm if additional** | **Technical accuracy** | **Other results** |
| --- | --- | --- | --- | --- | --- | --- | --- | --- |
| Hanauer et al., 2007 | Case detection for cancer registry | Cancer | USA | Hospital EMR: Unstructured free text pathology reports and clinical notes | **Rule Based Algorithm**: Text is processed by Case Finding Engine (CaFE), built from freely available open source components, including Java Server Pages and the Apache Tomcat Web server. Custom-made lists containing approximately 2,500 terms and phrases and 800 SNOMED codes. | **No algorithm reported.** Notes flagged for manual review. | Automated case identification Sensitivity = 100% Specificity = 85.0%. Clinical documentation Sensitivity = 100% Specificity = 73.7%. | Use of the system resulted in a considerable increase in the number of cases added to the cancer registry each month (p=0.003) |
| Baus, Hendryx and Pollard 2012 | EMR research | Hypertension | USA | EMRs from 11 primary care centres | **Keyword search** (incl. abbreviations, wildcard expressions and spelling mistakes) | **Codes but No algorithm:** Keyword or ICD code sufficient to define a case. | Performance not reported | 7% addition in patients identified when adding in free text search. |
| Coloma et al., 2013 | EMR research | Acute Myocardial Infarction | Netherlands, Italy, Denmark | Primary Care system (ICPC codes & text, Netherlands)Hospital system (ICD 9 & 10 codes & text; Denmark and Italy) | **Keyword Search,** extraction to UMLS terms. | **One code or keyword used** | ICPC code PPV 75% IPCI free text PPV 20% ICD-9 codes, best PPV 100% HSD free text 60% ICD-10 codes, best PPV 100% (no free text for comparison) |  |
| Thomas et al., 2014 | EMR research | Prostate cancer | USA | Hospital Pathology reports :Kaiser Permanente Southern California Medical Region | **Keyword Search**: KPSC Clinical Information Extraction System. Hierarchical keyword search to identify cases of prostate adenocarcinoma. | **Text Only** combined several keywords to identify cases | PPV = 99.1 %, Sensitivity = 99.1% Specificity = 99.9 % Accurate extraction of additional variables = 97.6 %. |  |
| Bellows et al 2014 | EMR research | Binge Eating Disorder (BED) | USA | Hospital EMR (VA) containing patient demographics, diagnostic codes, vital signs, medications, and narrative clinical notes | **Rule Based Algorithm:** For NLP, approach developed starting with the initial terms and phrases found in the EDNOS patient notes, search was for physician diagnosis of BED. A customized algorithm, similar to ConText, [35] was used to identify instances when a term or phrase was found in a clinical note indicating a diagnosis of BED was negated, hypothetical, historical, given to someone other than the patient, or included in informative text. | **Text extraction algorithm only** | Correct classification of having BED in 90.7% of cases, as not having BED in 96.6% of cases. Sensitivity of 96.2% | No significant differences in patient characteristics between BED and EDNOS. |
| Gundlapalli et al., 2008 | EMR research | Inflammatory Bowel Disease | USA | Hospital and Primary Care EMR (VA). Clinical notes from primary care, specialty clinics and the emergency department | **Rule Based Algorithm:** MedLEE coupled with a negation algorithm called NegEx. (search strings are mapped to semantic lexicon containing concepts from UMLS and assigned concept unique identifier, category and modifier). | **Text extraction algorithm only** | Sensitivity = 86% (77-92) Specificity = 99% (99-99) PPV 43% and NPV = 100% |  |
| Alemi et al., 2012 | Epidemic surveillance | Influenza | USA | Military Medical Treatment Facility EMR: containing laboratory orders, unstructured notes on reason for appointment, ICD-9 codes | **Bayesian probability approach** – a naive Bayes model for processing text of reasons for appointments and which accounts for dependence among words. Misspellings and abbreviations are accounted for. All the words in patients reason for appointment were assessed | **Text extraction algorithm only** | For the independent model: sensitivity 0.66 and specificity 0.48. For the dependent model (allowing correlations between words): sensitivity 0.49 specificity 0.64. PPV 0.61 |  |
| Friedlin et al., 2008 | Epidemic surveillance | MRSA | USA | Electronic laboratory reporting system (microbiology culture results reported as unstructured, freeform text) | **Rule Based Algorithm:** Regenstrief Extraction Tool (REX) – a rule-based NLP system written in Java. Modular design which can be easily adapted. | **Text extraction algorithm only** | F-measure = 0.9989 |  |
| Jones et al., 2012 | Epidemic surveillance | MRSA | USA | Veterans administration microbiology data: semi-structured, free-text form, “human-readable” as microbiology report | **Rule Based Algorithm:** NLP system developed in Apache Unstructured Information Management Architecture (UIMA), pipeline of processes, taking advantage of native structure for semantics and relationships. Rule based system: section identification, organism identification, susceptibility detection, MRSA inference. | **Text extraction algorithm only** | In comparison to electronic data, Sensitivity 100% Specificity 99.9% PPV 99.9% In comparison to expert-reviewed data Sensitivity 99.2% Specificity 99.4% PPV 97.9% |  |
| Yang et al., 2009 | Case Detection NOS | Obesity | USA | Hospital Discharge Reports (I2B2 challenge data) | **hybrid algorithm:** term matching, sentence filtering, sentence labelling, result integration. | **Text extraction algorithm only** | Macro F measure Textual task 0.81 Intuitive task 0.63 |  |
| Childs et al., 2009 | Case Detection NOS | Obesity | USA | Hospital Discharge Reports (I2B2 challenge data) | **Rule Based Algorithm:** ClinREAD (http://www.rocketsoftware.com/products/ rocket-aerotext). The final system contained 281 rules, 33 elements, 567 features, and 20 actions | **Text extraction algorithm only** | Microaveraged F-scores: 0.98 Textual analysis 0.96 Intuitive analysis |  |
| Farkas et al., 2009 | Case Detection NOS | Obesity | USA | Hospital Discharge Reports (I2B2 challenge data) | **Rule Based Algorithm:** Components: Keyword/Excluding Term Selection, Irrelevant Contexts, negation, uncertainty detection, intuitive terms and biomarker expressions | **Text extraction algorithm only** | Textual Results F-macro 84% on training set and 76% on test set. Intuitive Results: F-macro 82% on training set and F macro 67% on test set. |  |
| Mishra et al., 2009 | Case Detection NOS | Obesity | USA | Hospital Discharge Reports (I2B2 challenge data) | **Rule Based Algorithm:** text preprocessing, identification of keywords and associated assertion types, document scoring and classification. Also used Negex. | **Text extraction algorithm only** | Macro F-measures of 0.74-0.76 on training set. Macro F-measure for obesity 0.4917 |  |
| Solt et al., 2009 | Case Detection NOS | Obesity | USA | Hospital Discharge Reports (I2B2 challenge data) | **Rule Based Algorithm:** Abbreviation Resolution, identify discharge summary elements, context aware classification of Y/N/Q from dictionary terms | **Text extraction algorithm only** | Obesity: Textual F1-Macro 0.49, Intuitive F1-macro 0.97 Average Textual F1-macro 0.80, Intuitive F1-macro 0.67. |  |
| Ware et al., 2009 | Case Detection NOS | Obesity | USA | Hospital Discharge Reports (I2B2 challenge data) | **Rule Based Algorithm:** Feature extraction, concept context, and rules | **Text extraction algorithm only** | Macro scores, textual judgement, obesity: Precision: 0.73 Recall: 0.49 F: 0.49 System average Precision: 0.83 Recall: 0.75 F: 0.78 Macro scores, intuitive judgement Precision: 0.98, obesity: Recall: 0.98 F: 0.98 System average Precision: 0.64 Recall: 0.64 F: 0.64 |  |
| South at al., 2008 | Epidemic surveillance | Influenza- like illness (ILI) | USA | Hospital (VA) EMR containing free text notes from clinical encounters, incl. chief complaint string; emergency department notes; nursing or nurse triage notes; routine clinic visit notes | **Rule Based Algorithm:** Simple text classifier based on string matching for concepts mapping to UMLS. Coupled with negation algorithm NegEx.. Search using keywords for case definition defined by clinical expert**.** | **Rule Based Algorithm:** Document sources combined to get best result. | Specificities always > 95%. Sensitivity = 89% based on the full note corpus. AUROC = 92% based on full corpus |  |
| Turchin et al., 2005 | estimation of incidence | Diabetes | USA | Primary Care Research Patient Data Registry – containing lab results, physician notes, radiology reports, administrative/billing records | **Keyword Search:** DITTO (Diabetes Identification Through Textual Element Occurrences) searches for the presence of words, word roots or groups of words, including “diabet” etc. Strings excluded if negated. | **Rule based algorithm**: Patients with two sentences with diabetes word tags but without negative qualifiers were considered to have diabetes. | Sensitivity: 96.2% Specificity: 98.0% |  |
| Sieyfried et al 2010 | Clinical study recruitment | Depression | USA | Hospital EMR: CareWeb: Patient encounters, problem lists, medication data, pathology and radiology reports encoded as free text, | **Keyword Search:** Use of a medical record search engine, EMERSE [32]. “Bundles”, or groups of search terms (keyword list), can be created to perform standardized searches of patient lists; include case-sensitive searches and wildcard matches | **Rule Based Algorithm:** 4 criteria were searched for in text and combined. | EMERSE vs manual chart abstraction: As accurate (p>0.05) Significantly faster (p=0.03) |  |
| Mishra 2012 | Clinical decision support | Diabetes | USA | Hospital Discharge summaries from I2b2 shared task. | Rule Based Algorithm: ConText performs: 1) concept extraction, 2) concept summarisation 3) measurement extraction; 4) classification . [35] | **Rule Based Algorithm:** Classification on the basis of 1) discourse indicators only 2) medication indicators only 3) Combination. | For positive classification, F measure = 0.9865 Combined with negative and unknown classification, F measure 0.9865 |  |
| Fiszman et al., 2000 | Clinical decision support | Pneumonia | USA | Chest X-ray reports from Utah Hospital. | Hybrid Algorithm: SymText [97] A syntactic component is implemented as a set of augmented transition network grammars, semantic component consists of three different Bayesian networks. | **Rule based algorithm** applied to SymText output to determine presence or absence of pneumonia concept. | SymText: For acute bacterial pneumonia: Recall 0.95 Precision 0.78 Specificity 0.85 |  |
| Wilke et al., 2007 | EMR research | Diabetes mellitus (DM) | USA | Hopsital EMR: Marshfield Clinic Personalized Medicine Research Project (PMRP) DNA biobanks, EMR contains events and encounters (ICD codes), office notes, operative reports and discharge summaries, medication alerts | **Keyword Search:** FreePharma NLP software used to extract medications. | **Rule Based Algorithm:** Hierarchical Algorithm utilized diagnostic code, clinical laboratory data (glucose level) or medication history to identify subjects with DM. | Sensitivity Diagnostic codes combined with lowest HbA1c rate: 55.6% highest HbA1c rate: 97.0% | This algorithm yielded an estimated prevalence of 24.2% for diabetes mellitus in adult subjects aged ≥50 years. |
| Graiser et al., 2007 | EMR research | Follicular lymphoma | USA | Hospital EMR: Cancer registry data, electronic medical records (EMR), laboratory, administrative, pharmacy, and other clinical data | **Keyword search:** Text-string searches in anatomical pathology reports, and all medical records. Query using the UMLS Metathesaurus Concept Search was performed to obtain synonyms for follicular lymphoma. NEAR function | **Rule Based Algorithms:** Different queries were tried (12 in total), using codes, text or a combination. | Ranges: Sensitivity 6.7-93.5 Specificity 24.8-99.3 Best performing (Q4): Sensitivity 90.0 Specificity 85.9 |  |
| Valkhoff 2014 | EMR research | Upper GI bleeding | Netherlands, Italy and Denmark | 4 Hospital/Primary Care EHR systems using ICD 9 (2 systems; HSD and ARS), ICD 10 codes (Aarhus), and (International Classification of Primary Care) ICPC codes (IPCI). 2 primary care systems: clinical notes, letters from specialists. 2 hospital systems administrative database and discharge letters. | Free text **keyword search**; strings in Dutch and Italian. Extraction to UMLS terms. | **Rule Based Algorithm:** Cases identified by code from code list or from text string in free text. | 1) IPCI database: PPV was 22% for free text and 21% for ICPC codes 2) HAS database: PPV 91% for ICD 9 codes, and 47% for free text 3) ARS database: PPV 72% (ICD 9 codes) 4) Aarhus database PPV 77% (ICD-10 codes) |  |
| Peissig et al 2012 | EMR research | Cataract | USA | Hospital EMR: Personalised Medicine Research Project, includes diagnoses, procedures, medications, clinical notes, radiology, laboratory, and clinical observations in ICD 9 codes | **Rule Based Algorithm:** MedLEE was used, which parses narrative documents and outputs XML documents using UMLS CUI. | **Rule Based Algorithm:** Cataract and subtype identified in code first. Then text searched to find additional cases. If subtype was unclear then linked eye exam images were examined using optical character recognition. | PPV of 95.6% NPV of 95.1% |  |
| DeLisle et al., 2013 | Epidemic Surveillance | Acute Respiratory Infections | USA | Hospital EMR: Chest imaging report, Clinical record incl. clinical notes, ICD-9 codes, and prescriptions | **Rule Based Algorithm:** cTAKES NLP system (reported in Savova 2010 http://www.ohnlp.org) | **Rule Based** algorithms, including codes, text strings, and prescriptions. | Precisions ranged 64–86%; Sensitivities ranged 58–75%. |  |
| Hripcsak 2009 | Epidemic Surveillance | Influenza-like illness and gastrointestinal infectious disease | USA | Ambulatory EHR from community health centres – structured data and narrative text. | **Rule Based Algorithm:** MedLEE natural language processor, based on a semantic grammar and a lexicon, produces an XML-encoded set of findings and modifiers | **Rule Based Algorithm:** Queries based on structured data and narrative data, compared to influenza isolates. Queries were adjusted manually to visually maximize the signal-to-noise ratio of plotted time series | Influenza: ROC area = 0.993 Sensitivity = 1.000 (95% CI 0.822–1.00), Specificity = 0.986 (0.983– 0.989) PPV = 0.234 (0.157– 0.333) NPV = 1.000 (0.999 –1.000). Gastrointestinal infection: ROC area = 0.944 Sensitivity = 0.929 (95% CI 0.642– 0.996) Specificity = 0.959 (0.948–0.967), PPV = 0.141 (0.080–0.233) NPV = 0.999 (0.997–1.000). |  |
| Van Lier 2014 | estimation of incidence | Varicella (chicken pox) | Netherlands | Integrated Primary Care Information database, consisting of codes, clinical findings, lab results, prescriptions, referrals and letters. | **Manual review** of codes and free text | **Rule Based Algorithm:** All patients with diagnosis ICPC-code 72(=varicella/chickenpox) and all patients with chickenpox (Dutch: ‘waterpokken’), varicella or VZV in the free text fields in the medical journal were considered to be potential varicella cases. | Not assessed | The overall incidence of GP-consultation due to varicella per 100,000 person-years was at least 281(95%CI 268–294). |
| Suijkerbuijk et al., 2011 | estimation of incidence | Chlamydia | Netherlands | Primary Care records including ICPC codes, lab results in free text and prescription of antibiotics. | **Keyword search** with string “chlam” | **Rule Based Algorithm:** Cases defined on ICPC codes, both chlamydia specific and higher order codes if the treatment fitted a chlamydia diagnosis. | Not reported | Overall incidence ranged from 103.2/100,000 to 590.2/100,000 |
| Hanauer et al.,2014 | Clinical study recruitment | Disorders of sex development | USA | Hospital EHR: Structured billing codes and free text billing documentation | Keyword Search: Search engine software (EMERSE). | **Rule Based Algorithm:** Combination of structured billing ICD-9 codes as an initial filtering strategy followed by keywords applied to the free text clinical documentation | Performance not reported portability reported | Traditional approaches yielded 14 and 28 patients with DSD, at two institutions the informatics approach yielded 226 and 77 patients, respectively. |
| Li Li et al., 2008 | Clinical study recruitment | Ischemic stroke | USA | Hospital EMR : Clinical Data Warehouse (CDW) from hospital containing diagnoses, procedures, discharge summaries, laboratory tests, and pharmacy data | **Rule Based Algorithm:** Medical Language Extraction and Encoding System (MedLEE) used to process the narrative discharge summaries. MedLEE generates structured, computer-recognizable, XML-coded concepts using semantic classes for problem, finding, body location, certainty, degree, region etc. | **Rule Based Algorithm:** Combination of codes and text put in algorithm chosen by clinical experts. | Medlee: True Positive =28 (56%) False Positive =22 (44%) ICD9: True Positive=27 (90%) False Positive =3 (10%) | NLP processed discharge notes provided richer information than ICD9 codes for clinical trial pre-screening, identifying 702 more eligible patients than using the ICD9 query alone. |
| Schmickl et al., 2011 | Clinical study recruitment | Chronic Obstructive Pulmonary Disease | USA | Hospital EMR: Mayo Clinic Life Sciences System (MCLSS) warehouse, including ICD9 codes, admission notes and reports. | **Keyword Search:** Electronic free-text search engine; E-screening tool queries the free-text sections in the admission notes of the EMR for any of the following terms: COPD, COPD exacerbation, respiratory failure, hypercapnia, emphysema, chronic bronchitis, home O2 | **Rule Based Algorithm:** Patients excluded if met ineligibility criteria and then NLP searched the record for COPD related terms. | 100% negative predictive value 72% positive predictive value | Time-saving of about 40 min per day (76%) compared to nurse manual review. |
| Ludvigsson et al., 2013 | Case detection NOS | Coeliac disease | USA | Hospital EMR: (Mayo Clinic) including ICD-9 codes, clinical notes | **Rule Based Algorithm:** NLP formed of dictionary comprising named entities of interest, decided on by clinical experience, incl. symptoms. Algorithm contained pipeline to context determination – time, subject and negation. The optimal number of hits assessed. | **Rule Based Algorithm:** Compared 16 keywords in various combinations to ICD codes combinations. | Keyword best performance: Sensitivity = 72.9% Specificity = 89.9% PPV in unselected population: 4.2% ICD code best performance: Sensitivity: 17.1% and Specificity 88.5%, PPV 0.9% |  |
| Pakhomov et al., 2007 | Case detection NOS | Angina pectoris | USA | Hospital EMRs: Billing records of in- and out-patients, and dictated, transcribed consultation notes structured in conventional sections. | **Rule Based Algorithm:**  Text Analysis system created at the Mayo Clinic based on keywords supplemented with wildcard characters. Algorithm for negation similar to NegEx | **Rule Based Algorithm:** ICD code search and text search compared to standardised questionnaires (Rose questionnaire) | Unspecified chest pain: EMR-NLP true positive rate = 62% (95%CI:55–67) Diagnostic codes true positive rate = 51% (95%CI:44–58) (p<0.001). Exertional chest pain:EMR-NLP true positive rate = 71% (95%CI:61–80) Diagnostic codes true positive rate = 62% (95%CI:52–73) (p=0.10)Rose angina: True positive rate = 88% (95%CI: 65–100) |  |
| Cano et al., 2009 | EMR research | Obesity and comorbidities | USA | Hospital Discharge Summaries | **Rule Based Algorithms**: LingPipe (http://alias-i.com/lingpipe) used on three feature sets:1) character 5-grams 2) Bag-of-Words representation enriched with small set of additional features. 3) Pruned Lexicon | **Multinomial Logistic Regression** Classifier (maximum a posteriori point estimates with Laplace priors, using stochastic gradient descent). | Macro Precision 1) 0.956 2) 0.567 3) 0.763 Macro Recall 1) 0.446 2) 0.484 3) 0.457 Macro F-measure 1) 0.451 2) 0.506 3) 0.464 |  |
| Ananthakrishnan et al., 2013. | EMR research | Inflammatory Bowel Disease | USA | EMRs of tertiary referral hospitals incl: outpatient notes, discharge summaries, operative notes, radiology, endoscopy, pathology | **Rule Based** System Used CTAKES to extract text (reported in Savova 2010 http://www.ohnlp.org) - modular system of pipelined components combining rule-based and machine learning techniques. | **Penalized logistic regression:** Combined algorithm with text, prescriptions and codes. Variables chosen with logistic regression. | AUC = 0.89 for Crohn’s disease AUC = 0.86 for ulcerative colitis. | Addition of NLP narrative terms to model resulted in classification of 6–12% more subjects with the same accuracy compared to codes alone. |
| Carroll et al., 2012 | EMR research | Rheumatoid Arthritis | USA | Hospitals: Vanderbilt, Northwestern and Partners Healthcare EMRs. Codified data and clinical narratives | **Rule based algorithm** Narratives were searched using NLP system to map medical terminology from free text to controlled vocabularies (UMLS). Systems used were HITEx NLP system, Knowledge Map Concept Identifier and SecTag MedEx, which produced RxNorm-encoded medications | Logistic regression. Case detection algorithm combined codes and text (reported in Liao et al.)– classification using logistic regression | Portability of algorithm between 3 different EMRs. Developed on partners, tested on Vanderbilt and Northwestern EMRs Vanderbilt PPV = 95% Sensitivity = 57% AUROC = 95% Northwestern PPV = 87% Sensitivity 60% AUROC = 92% |  |
| Liao et al, 2010 | EMR research | Rheumatoid Arthritis | USA | Hospital EMR with codified (ICD-9 codes) and narrative data (health care provider notes, radiology reports, pathology reports, discharge summaries, operative reports | **Rule Based Algorithm:** Health Information Text Extraction (HITEx) system | Classification algorithms developed on combined codes and text information from the records, using **penalised logistic regression** with adaptive lasso procedure. Optimal penalty parameter was determined based on the Bayesian information criterion | Complete algorithm (specificity set at 97%) PPV = 94% (95%CI: 91-96% Sensitivity = 63% (51-75) |  |
| Xia et al 2013 | EMR research | Multiple sclerosis | USA | Hospital EHR: containing demographics, billing codes for diagnoses and procedures, laboratory results, electronic prescriptions, clinical encounter notes, imaging reports | **Rule Based Algorithm:** Using cTAKES, narrative variables on symptoms, signs, medications, MRI reports, and neurologist’s impression and treatment plan were extracted (ctakes.apache.org) | LASSO penalized **logistic regression** models with Bayesian Information Criterion to select variable for predicting MS diagnosis. | Identification of patients with 95% specificity threshold: AUC = 0.958 For a specificity of 95%: Sensitivity, 83% PPV = 92% NPV = 89% |  |
| Huang et al., 2014 | EMR research | Depression | USA | Hospital/Community EMR systems, incl. ICD-9 codes, RxNorm Prescription codes, Procedure codes, pathology reports, radiology reports, and transcription reports. | **Rule Based Algorithm:** Optimized version of the NCBO Annotator with a set of 22 clinically relevant ontologies; remove ambiguous terms, flag negated terms and terms attributed to family history. Terms are normalised to CUIs. ICD-9 codes, RxNorm codes, and CPT codes also normalized to Concept Unique Identifiers (CUIs) from the Unified Medical Language System (UMLS) Metathesaurus. | LASSO **Logistic regression** models trained to predict diagnosis of depression, response to treatment and severity. | AUC for diagnosis: 0.8 (95% CI 0.784-0.815) at 90% specificity, sensitivity is 50% at time of diagnosis. |  |
| DeLisle et al., 2010 | Epidemic Surveillance | Pneumonia | USA | Hospital EMR (Veterans Administration (VA)): Outpatient veterans records | **Rule Based Algorithm:** National Library of Medicine UMLS Metathesaurus search tool [106] which maps concepts to concept unique identifiers. Then examined all of the UMLS-supplied lexical variants and semantic types related to ARI to build the final list of strings. NegEx version 2 algorithm to check for negation. | Practicing clinicians systematically reviewed and adjucated structured or semi-structured EMR parameters for inclusion. Parameters retained in model using backward elimination logistic regression | Best Algorithms: ICD 9 codes, or text, and temp >37.8c: Sensitivity 100% Specificity 99.7% PPV 34%, AUC 100% ICD 9 codes, and text, and temp > 37.8c: Sensitivity 71% Specificity 99.9% PPV 68%, AUC 85% | Inicidence of 1.8%. Cough was the most commonly documented symptom of ARI (88% of cases), followed by fever/chills/night sweats (58%) and sore throat (45%). Eight (8) percent of ARI patients were febrile at the time of the encounter. ARI cases were younger than the study population |
| Zheng et al., 2014 | Epidemic surveillance | Acute respiratory infection | USA | VA Hospital EMR, ICD codes, prescriptions, and clinical notes. | **Rule Based Algorithm:** As reported in De Lisle 2010, NLP performed with National Library of Medicine UMLS Metathesaurus search tool combined with NegEx detection algorithm. | **Logistic Regression** Previously developed ARI case-detection algorithms (CDAs) | Sensitivity ranged 63-99% Specificity ranged 89-99% PPV ranged 13-54% AUROC ranged 78-94% |  |
| Xu et al., 2011 | EMR research | Colorectal cancer (CRC) | USA | Hospital EHRs (Vanderbilt University), discharge summaries, clinical communications, clinical forms, radiology notes, pathology notes, patient summary lists, ICD-9 codes, CPT codes | Rule Based Algorithm: MedLEE NLP system | **Rule Based Algorithm compared to Machine Learning:** For document level classification, 2 approaches tried: heuristic rule-based approach, compared to support vector machine learning. For patient level classification, Rule based methods, and Four types of machine learning were tried: Random Forest, Ripper, Support Vector Machine, and Logistic Regression. | Best: document level concept identification: F-measure = 0.996 patient level case detection: F-measure = 0.93 |  |
| Afzal et al., 2013 | EMR research | Asthma in children | Netherlands | Primary Care Database: coded data and anonymized data on patient demographics, symptoms and diagnoses, clinical findings, referrals, laboratory findings, and hospitalization | Machine learning method (bag of words representation), with chi-square feature selection. Dutch assertion filter for negation | **Machine learning** algorithm (hierarchical multi-class classification); combination of codes and text. RIPPER – rule learning algorithm, with five fold cross validation. | PPV = 0.66 sensitivity = 0.98 specificity = 0.95. |  |
| Carroll et al., 2011 | EMR research | Rheumatoid Arthritis | USA | Hospital Medical Centre records (de-identified synthetic derivative), formed of ICD-9 codes, NLP results turned into UMLS, and medications | **Machine Learning**  NLP identified section of EMR where text belongs, and then processed to return concept unique identifiers, plus negation status. Medication attributes were generated from medications found by MedEx, an NLP medication extraction tool, and filtered to those instances containing at least one of the following: dose, route, amount, or frequency. | **Machine Learning**  Support Vector Machines combined text medication and codes to identify cases. | Naïve system Precision = 93.3% Recall = 79.7% Refined system Precision = 93.7% Recall = 85.8% |  |
| Afzal et al., 2013 | EMR research | Hepatobiliary disease; Acute renal failure | Netherlands | Primary Care Database, containing symptoms, physical examination, assessments, and diagnoses, prescriptions and indications for therapy, referrals, hospitalizations, and laboratory results | **Rule Based Algorithm:** Electronic search of text, including any words, misspellings, or part of the words relevant to the case definition. Assertion filter screened for speculation, negation and alternatives keywords. Following screening, remaining words treated in bag-of-words representation. | Machine learning algorithms to combine features. Decision tree learners (C4.5 and MyC); support vector machines (libsvm); RIPPER; and MetaCost to make a cost sensitive algorithm | Best algorithms: Hepatobiliary disease: MyC Sensitivity 0.95 Specificity 0.56 Acute Renal Failure: C4.5 Sensitivity 0.86, Specificity 0.77 Ripper Sensitivity 0.89, Specificity 0.59 |  |
| Karnik et al., 2012 | EMR research | Atrial fibrillation and/or atrial flutter | USA | Hospital EMRs, text fields taken from clinic office notes, interpretations of radiological findings and hospital discharge summary. Also used ICD9 diagnoses, laboratory values, vitals, procedures and prescriptions | **Rule Based Algorithm:** Mapped drugs, chemical, disease and symptom phrases in the textual data to UMLS vocabulary using MetaMap, with the following options: word sense disambiguation, ignore word order and negation detection | **Machine learning** methods: naïve bayes, support vector machines (SVM), logistic regression and random forests Codes only, text only and combination all tested. | Best performances: Text only; random forest: Precision: 58% Recall: 62.7% F: 60.1% Codes Only; random forest Precision: 59.8% Recall 61.7% F: 60.6% Codes and Text: Random Forest, Precision: 60% Recall 60% F: 60`% |  |
| Tsui et al., 2011 | Epidemic surveillance | Influenza | USA | Emergency department dictated notes | **Rule Based Algorithm:** MEDLEE and Topaz (determines the presence, missing, or absence (negation) of 51 findings expected in influenza). Pipeline: module 1 looks for relevant conditions, module 2 determines negation, history, hypothetical, non-patient, module 3 assigns value of present, absent or missing. | **Bayesian network model** of medical diagnosis and natural language processing comprised of flu symptoms, findings, and lab tests. In one model, symptom and sign and conditional probabilities built by clinical expert. 2^nd^ algorithm was machine learning Expectation Maximization-Maximum-A-Posteriori algorithm. | Expert model AUROC = 0.956 (95% CI: 0.936-0.977) EM-MAP model AUROC = 0.973 (95% CI: 0.955-0.992). |  |
| Love et al., 2011 | Case detection NOS | Psoriatic arthritis | USA | Hospital EMR: containing Clinic notes, billing codes, encounter information, provider information, laboratory values, and radiology reports | **Keyword Search:** Terms from clinic notes were identified in medical records using the standard SQL query language | **Machine Learning**: 3 random forest algorithms were trained using coded, narrative, and combined predictors. | PPV = 93% (95%CI 89%-96%) Sensitivity = 79% (77%-82%), Baseline (single billing code) PPV = 57% (52%-61%) |  |
| Castro 2015 | EMR research | bipolar disorder | USA | Hospital EMR: ICD-9 codes, medications, procedures, lab values and unstructured clinical notes | **Rule Based Algorithm:** Hitex. Terms extracted from narrative with NLP using Hitex | **Logistic regression** classifier with LASSO procedure. Optimal penalty parameter was determined on the basis of the Bayesian information criterion. **Rule based classifier** also developed on only coded information. | Log Reg algorithm: NLP model: PPV = 0.85 Best coded model PPV = 0.79. Rule based algorithm: NLP PPV = 0.86, best coded PPV = 0.84 |  |
| Chapman et al., 2004 | Epidemic surveillance | Fever | USA | ER clinical narrative (text only) | **Naïve bayes** classification system plus NEgex | **Text only:** Keyword HP algorithm (rule based), Keyword CC algorithm (rule based) CoCo algorithm naıve Bayesian classifier with a probabilistic model | Keyword HP Sensitivity 0.98; Specificity 0.89; Keyword CC; Sensitivity 0.61; specificity 1.0; sensitivity CoCo 0.57; specificity 0.95 |  |
| Eland et al., 2001 | estimation of incidence | priapism | Netherlands | Primary Care records including ICPC codes, and free text | **Keyword search** | Search for code or text, followed by **manual review** | not reported |  |
| Elkin et al., 2008 | Epidemic surveillance | Pneumonia | USA | radiological reports (chest x ray or CT scans | **Rule Based Algorithm:** Multi-threaded Clinical Vocabulary Server, codeing output into snomed terminology | **Rule based** (text only) | With uncertaintly, sensitivity = 100%, specificity = 90.3% PPV = 70% Without uncertaintly, sensitivity = 100%, specificity = 97.99%, ppv = 97.44% |  |
| Koopman et al., 2009 | estimation of incidence | facial pain | Netherlands | primary care records including ICPC codes and free text | **Keyword search** | **Manual review** | PPV of first step was 57.6% |  |
| McPeek et al., 2013 | EMR research | venous thromboembolism | USA | Hospital EMR, ICD-9 codes, clinical notes, problem lists | **Rule Based Algorithm:** KnowledgeMap Concept Identifier (KMCI) | **Rule based algorithm** | Codes alone, ppv = 69.0%; NLP = sensitivity 95.1%, PPV 90.0%, F measures, 0.925; second sample: codes alone 70.3%, NLP sensitivity 95.3%, PPV 84.7%, F measure 0.897. |  |
| mehrabi et al., 2013 | surveillance for prevention | pancreatic cyst | USA | Hospital EMR | **Rule based algorithm** Unstructured Information Management Architecture (UIMA) pipeline, plus REX algorithm plus negex (outputs UMLS ) | **Text only** (rule based algorithm combines text outputs) | precision = 98.9, recall 95.7%, f measure 97.57% |  |
| mendonca et al., 2005 | Epidemic surveillance | neonatal pneumonia | USA | Hospital EMR including radiology, pathology and mircobiology reports, chart reviews | **Rule based algorithm**: MedLEE | **Rule based algorithm** (text only) | sensitivity 71%, specificity 99% but PPV was only 7.5% |  |
| pakhomov et al., 2005 | Clinical study recruitment | congestive heart failure | USA | Hospital EMR clinical notes | **Rule based algorithm** for extraction, with output in MeSH and HICDA classification schemes | **Machine learning** (Text only) naïve bayes and perceptron neural network | Study reports accuracy (true designations divided by all examples) and recall of positive samples. Mean naïve bayes accuracy = 82%, mean perceptron = 86.5%. |  |
| pakhomov et al., 2007 | EMR research | heart failure | USA | Hospital EMR including ICD-9 codes and clinical notes | **Rule based algorithm** | Text only **naive bayes** model | NLP sensitivity 81.6%, specificity 97.8%, ppv = 49% Naïve bayes: sensitivity 56%, specificity 96% and PPV 82% |  |
| roch et al., 2015 | surveillance for prevention | pancreatic cyst | USA | Hospital EMR clinical reports | **Rule based algorithm**: Unstructured Information Management Architecture (UIMA) | Text only **rule based algorithm** | sensitivity 99.85% specificity 98.8% |  |
| savova et al., 2010 | EMR research | peripheral arterial disease | USA | radiology notes | **Rule-based algorithm** CTAKES | Text only **rule based algorithm** | sensitivity positive cases 0.93, specificity, 0.99; PPV = 0.99 and NPV = 0.93 |  |
| Tian et al., 2015 | surveillance for prevention | venous thromboembolism | Canada | Hospital EMR. imaging narrative reports | **Rule based algorithm** | Text only **rule based algorithm** | DVT classifier: sensitivity = 94%, specificity = 96%, PPV = 73%. PE classifier - sensitivity = 94%, specificity = 96% PPV = 80% |  |
| Wu et al., 2013 | EMR research | asthma | USA | Hospital EMR with ICD codes and text | **Rule-based algorithm** CTAKES | **Machine learning system compared to rule based system**. Decision tree algorithm | ICD 9 codes: sensitivity: 30.8, specificity: 93.2%, PPV 57.1%, NPV 82.2, F score 40.0. Best NLP system sensitivity 84.6%; specificity 96.5%, PPV 88.0%, NPV 95.4%, F score 86.3% |  |
| Yadav et al., 2013 | EMR research | acute orbital fracture | USA | CT imaginng reports from hospital ED | **Rule-based algorithm** MedLEE (outputting UMLS), followed by Waikato Environment for Knowledge Analysis (WEKA) 3.7.5 and Predictive Miner 6.6. | **Rule based algorithm** classification and decision trees (text only) | NLP output performance: sensitivity 0.933, specificity 0.969, PPV 0.816, NPV 0.990 |  |
| Ye et al., 2014 | EMR research | influenza | USA | ED reports | **Rule based algorithms:** Topaz and MedLEE | Outputs combined by 3 **bayesian networks**, 1 expert defined and 2 machine learning. (Gold standard was lab test results) | recall (sensitivity)Topaz 0.80, medlee 0.79, precision(ppv) topaz 0.85, medlee 0.90 specificity topaz 0.76 medlee 0.62 |  |
| Yu et al., 2015 | EMR research | Rheumatoid arthritis and coronary artery disease | USA | hospital EHR | AFEP - **rule based algorithm** with UMLS output | Combined codes and text using **logistic regression** | Expert curated model AUC for RA = 0.938 and for CAD = 0.929. AFEP selected feature model AUC for RA = 0.951 and for CAD = 0.929. RA For a specificity of 95%, sensitivity was 0.70 and PPV was 0.795. CAD for a specificiyt of 95%, sensitivity was 0.71 and PPV was 0.903. |  |
| Zeng et al., 2006 | EMR research | Asthma and COPD | USA | Hospital discharge summaries | **Rule based algorithm**: Hitex. Maps to UMLS | **Rule based algorithm** for combining output | precision codes: 82.3, text only 82.3, text and code 87.4%. Sensitivity codes 72.5; text 76.7; codes and text 92.4. Specificity codes 90.9; text 87.0; codes and text 81% |  |
| Tanushi et al., 2014 | surveillance for prevention | hospital acquired urinary tract infection | Sweden | Hospital EHRs | **Keyword search** for uti specific bacteria | **Rule based algorithm** combining text and coded fields | recall in new data: 0.6, precision 0.98, f-score .74, specificity 0.999 npv 0.98. |  |
